# A School-Based Educational Intervention for School-Aged Children and Caregivers about Rational Use of Antibiotics in Urban Areas of Shaanxi Province: A Study Protocol for a Randomized Controlled Research

**DOI:** 10.3390/ijerph15091912

**Published:** 2018-09-03

**Authors:** Yu Zhang, John Kabba, Jie Chang, Wenjing Ji, Shan Zhu, Jiale Yu, Sen Xu, Yu Fang

**Affiliations:** 1 Department of Pharmacy Administration and Clinical Pharmacy, School of Pharmacy, Xi’an Jiaotong University, Xi’an 710000, China; m15829229279@163.com (Y.Z.); fudimamy@hotmail.com (J.K.); jiechang@xjtu.edu.cn (J.C.); Wenjing_Ji@harvardpilgrim.org (W.J.); zhu1992220@163.com (S.Z.); yujiale@stu.xjtu.edu.cn (J.Y.); marsxs@stu.xjtu.edu.cn (S.X.); 2 Center for Drug Safety and Policy Research, Xi’an Jiaotong University, Xi’an 710000, China; 3 The Global Health Institute, Xi’an Jiaotong University, Xi’an 710000, China; 4 Shaanxi Center for Health Reform and Development Research, Xi’an Jiaotong University, Xi’an 710000, China

**Keywords:** antibiotics resistance, upper respiratory tract infection, rational antibiotic use, educational intervention, children, caregivers

## Abstract

(1) Background: Antibiotic resistance is an imperative public health issue globally. Major factors that are resulting in this trend are the irrational and excessive use of antibiotics. Children account for a greater population of antibiotics use, therefore, an educational intervention on the rational use of antibiotics for children and caregivers will be beneficial. (2) Methods: A randomized controlled and parallel group study of fifth grade children and their caregivers will be evaluated in four primary schools in Baoji and Weinan of the Shaanxi Province. Two primary schools will be randomly selected for the educational intervention and two schools will serve as a control group. In the intervention arm, educational interventions will be conducted among caregivers and their children. The intervention measures include interactive training sessions, booklets, and printed or electronic educational materials that will be given to the caregivers or the school-aged children. In the control arm, no interventions will be implemented. Baseline data collected from June, 2018 and the intervention will last for three months. Knowledge-Attitude-Practice (KAP) questionnaires will be used to evaluate the caregivers’ knowledge, their attitude, and their practice. Knowledge-Retention questionnaires will be used to assess the children’s knowledge about microbes, antibiotics, and hygiene. (3) Discussion: This study is a unique comprehensive intervention targeting both children and their caregivers. We hypothesize a decrease in the irrational use of antibiotics among the studied population. Hence, this result would provide evidence for policy makers and educational departments for the implementation of similar interventions on the rational use of antibiotics.

## 1. Introduction

Antimicrobials are considered as one of the greatest inventions of the twentieth century [1]. Since their advent, they have been widely used in clinical practice and have played a great role in preventing and treating the infectious diseases of humans [2,3]. Currently, they account for the most consumed medicines worldwide [4], and the issue of bacterial resistance due to their overuse is a pressing global health concern [5,6,7]. There is a direct correlation between antibiotics abuse and the emergence of bacterial resistance, which increases the risk of adverse reactions and disease burden on the health system [8,9,10]. There has being a global drive towards research on this issue from key research and health promoting institutions in recent times. In 2010, the “Lancet” reported on “super bacteria” that can resist almost all antibiotics [11], and the 2011 theme of World Health Day was to Combat drug resistance—No action today, no cure tomorrow [12]. In 2014, a global antibiotic resistance monitoring report found that antibiotic resistance exists in all regions, revealing that at any time, the world will reach the “post-antibiotics” era [13], and in 2015, reports in China showed that the detection rate of methicillin-resistant Staphylococcus aureus (MRSA) was 35.8% and that the detection rate of erythromycin-resistant Streptococcus pneumoniae was 91.5% [14].

Children account for a greater proportion of antibiotics use in China, especially for upper respiratory tract infections in which it is common for them to be given antibiotics [15,16]. Even though these infections are mostly viral in nature [17,18] and there is evidence-based clinical guidelines pinpointing their ineffectiveness against colds, coughs, and sore throats, which are all of the most common respiratory tract infections [19,20], many caregivers still expect doctors to prescribe antibiotics for their children [21,22,23]. This practice has resulted in adverse events and health consequences in the population [24]. For example, a study in China on childhood deafness amongst children under the age of seven showed that the unreasonable use of antibiotics accounted for 30% to 40% of all cases, while in some developed countries it only accounted for 0.9% [25]. It has also been reported that the use of antibiotics in children is closely associated with their caretakers’ cognition, concepts, and habits about antibiotics [26]. In their study, using the Knowledge, Attitude, and Practice (KAP) questionnaire, Panagakou SG et al. [27] reported that although 80% of parents think that upper respiratory tract infections are viral infections, 74% of parents still expect their children to be treated with antibiotics.

In response to the irrational use of antibiotics in children, the majority of studies have focused on educational intervention to improve the knowledge about antibiotics in both children and caregivers [28,29,30,31,32,33,34]. Numerous studies [35,36,37] have shown that health educational intervention is an important method that can be used to promote the rational use of antibiotics. Some of these interventions are in the form of: professional education, training programs, seminars, publicity, educational materials, and face-to-face education for health service providers, patients, the general public, caregivers, policy makers, and so on [38]. In order to promote the rational use of antibiotics in children, children and their guardians need to work together. From the perspective of children, schools are one of the main crowded places and infectious diseases are easily transmitted among students. Therefore, educating students to prevent the spread of infectious diseases and decreasing the incidence of infectious diseases in children can reduce the use of antibiotics. In addition, children are a new generation of antibiotic users and may be future prescribers, therefore their awareness of rational use of antibiotics from a young age will have long-term effects. From the perspective of caregivers, the expectations of caregivers has a great impact on doctor’s antibiotic prescribing patterns for children. Therefore, improving knowledge about rational antibiotic use among caregivers is important for the well-being of children. Gaps exist in the literature on educational intervention studies targeting both children and their caregivers in China, hence, our study will attempt to fill such gaps and if the feasibility of the delivery of such interventions can be demonstrated, future studies will be able to investigate using our protocol as a model. Therefore, we conduct this research with the aim to assess the effectiveness of an educational intervention to improve school-aged children’s knowledge about hygiene and antibiotic use and caregivers’ knowledge, attitude, and practice about the rational use of antibiotics for children. In addition, we will try to elicit the knowledge, attitudes, and practice patterns of caregivers who are using antibiotics for children and will disclose knowledge about hygiene and antibiotic use among school-aged children. 

## 2. Materials and Methods

### 2.1. Study Design

**Figure 1:**
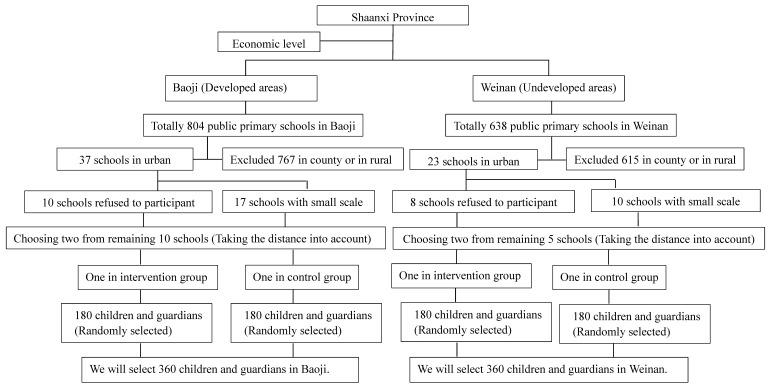
The sampling process.

We will conduct a multistage cluster randomized controlled trial at the school-level. Our study will be carried out in primary schools in the Shaanxi Province, western China. According to the economic level of the Shaanxi Province, two clusters were formed: developed areas, where we chose Baoji, and Weinan from the less developed areas. The chosen primary schools from both of the study sites met the following inclusion criteria: (1) Schools must be willing to take part in our study; (2) Schools should be in urban areas with enough teachers and students. In rural areas, most young people need to go out to work and earn a living, leaving the elderly to look after the children. Socio-demographic characteristics are quite different between rural and urban areas, so we only include urban primary schools. (3) Schools are theoretically matched and are comparable with each other in terms of school size and teaching level; (4) Must be public primary schools because private primary schools are mainly composed of students with higher family incomes. (5) Two schools in each city must be in different districts or in different streets. The distance between the two schools in each city must be more than 5 km. Based on the inclusion criteria and the referenced CONSORT (Consolidated Standards of Reporting Trials) statement for randomized trials [39], a final selection of two primary schools in each city will be made. In total, there are 804 public primary schools in Baoji and 638 public primary schools in Weinan. Among these schools, in Baoji there are 37 schools in urban areas and 767 in county or in rural areas. For the schools in urban areas, 10 schools are unwilling to participate in our study and 17 schools are small scale (which have less than three classes in the fifth grade or less than 1500 students in schools). Taking the distance between the two schools in the same city into account, we chose two schools from the remaining 10 schools. In Weinan, there are 23 schools in urban areas and 615 in counties or in rural areas. The sampling process was similar in Weinan and we have depicted it in Figure 1 (The sampling process). In each city, one primary school will be randomly selected for the intervention group and the other will serve as the control group. The study participants will be chosen from the class level and in every school we will select three to five classes from the fifth grade at random. Participants will be selected based on the inclusion criteria from the chosen classes and they will be blinded on their assigned group.

This study will be divided into three phases: pre-intervention, intervention, and post-intervention. In the pre-intervention phase, we will administer the KAP questionnaire and the Knowledge-Retention questionnaire to the caregivers and children to collect baseline data. Baseline data will be collected from June 2018. After baseline data is collected, we will implement health education interventions for the children and guardians, respectively, and it will last for three months. Immediately after the intervention, we will send questionnaires to the studied groups, that is, the children and caregivers. However, for school-aged children, we will again send the Knowledge-Retention questionnaire to them to evaluate the retention of knowledge one month after the intervention. Pre and post-intervention data will be compared between the intervention and the control groups, and we will then analyze the results to assess the impact of the interventions on antibiotics use among children.

### 2.2. Sample Size

Based on the previous study [40], the rate of irrational use of drugs by guardians for their children is 40% to 70%. Through our educational intervention, we hypothesized a 15% reduction in the rate of irrational drug use among children. We set α = 0.05, 1 − β = 0.9. We used software, Sample Size 2.0, to calculate the sample size, and 231 participants are required for the intervention and control groups. Taking into account the lost population, the invalid questionnaires, and the low response rate, we increased the sample size by 55%, to a total sample size of 720. We will select 180 school-aged students in every school. For each child included in our study, we will also invite their caregiver to participate in the study. In four primary schools, we chose 720 students and 720 caregivers. The sampling process is shown in Figure 1. We also invited teachers to help us to do our study. They are the bridge of communication between researchers and caregivers. Caregivers may not follow the orders from researchers, however most parents will follow tasks that are assigned by the teacher and, therefore, we will invite teachers to help us to implement a smooth intervention. In addition, they will help the researchers to distribute and collect the questionnaires from the children and caregivers. Before the intervention, we will give training to the teachers so that they will know more about our study and will clearly know their duty. In each class, we will invite 1 head teacher to cooperate with us.

### 2.3. Eligibility Criteria for Participants

1. We will include healthy children (aged 8–12) and caregivers who have the primary responsibility for the children and have normal understanding, cognitive, and communication skills.
2. Children and caregivers who volunteer to participate in this study must have lived in the sample area for more than a year and those with less mobility will be included. A written informed consent will be collected from all of the participants.
3. We will exclude children with severe liver, kidney disease, chronic diseases, and severe syndromes.
4. We will preclude caregivers who work in the health field.

### 2.4. Intervention Procedures

The purpose of the intervention is to change the knowledge, attitude, and behavior of guardians about the use of antibiotics for children with upper respiratory tract infections and, at the same time, to improve children’s knowledge about antibiotics use and to help them build awareness of preventing infection. We have created a WeChat (the most popular web-based social media tool in China) official account, through which we will push educational materials about the rational use of antibiotics to guardians. Only participants in the intervention group will have access to the information in the WeChat platform. Besides, participants are required to not share the information to others during the intervention period. For those caregivers who do not have WeChat accounts, we will distribute paper education materials separately, which will be the same as the electronic one. The educational materials are from the Center for Disease Control and Prevention in America [41]. Under the circumstance of China, we will translate the educational materials into Chinese and will make them easy to understand. We developed booklets about rational antibiotics use in children which cover the basic concept of antibiotics, antibiotic resistance, bacterial and viral infections, indications of antibiotics, and problems that can be encountered with the use of antibiotics and so on. The booklets will be taken home by the students. Both the students and the caregivers are required to study the booklet with commands from the teachers. Additionally, every month within a 3-month period, experts in the field will conduct a 1.5-h interactive training session for the children and their caregivers, and before each training session, we will issue them with the teaching materials. The content of the training session mainly includes four aspects: knowledge on microorganisms, the spread of infection, the prevention of infection, and the rational use of antibiotics for respiratory diseases. However, the training session for the caregivers will focus on the rational use of antibiotics and that for the children will concentrate on the knowledge of microorganisms, the spread of infection, and the prevention of infection. In the control group, both the children and caregivers will have no access to any intervention measures.

### 2.5. Outcome Indicators

For the caregivers, the primary outcome is the proportion of the caregivers who use antibiotics irrationally for their children. If one of the following situations exists, we will define it as irrational drug use: Guardians use antibiotics to prevent colds in children, increase or decrease the dosage of children on their own, reserve antibiotics at home or use residual antibiotics for children. The secondary outcome is scores of caregivers on knowledge, attitude, and behavior of antibiotic use for children. For the school-aged children, the primary outcome is the percentage of each of the questions that are contained in the questionnaires that are answered correctly. The secondary outcome is the scores of each section in the knowledge retention questionnaire.

### 2.6. Data Management 

During the period of pre-intervention and post-intervention, we organized the children and their guardians to complete the knowledge retention questionnaire and the KAP questionnaire, respectively, and for the illiterate participants, the researchers will read and explain the content of the questionnaire to them. Two different tools are used in this study: a knowledge retention and KAP questionnaire. Both questionnaires were developed based on published literatures [42,43,44,45,46] and were issued to the children and caregivers in one class before the study. According to the opinion of the children and caregivers, we revised the language of the questionnaire to ensure that the respondents can correctly understand the meaning of each item. In brief, the knowledge retention questionnaire has a total of 26 structured or semi-structured questions which can be divided into five sub sections: (1) Knowledge related to microbes, including differences between bacteria and viruses, and differences between bacterial infections and viral infections. (2) Hand hygiene, including the importance of washing hands frequently, when hands should be washed, and how to wash hands. (3) Respiratory hygiene, including the spread of infectious diseases through coughing or sneezing, and how to prevent the spread of bacteria when sneezing. (4) Food hygiene, including the problems of food contamination and cross-contamination and disposing leftovers and deteriorated foods. (5) Knowledge related to antibiotics, including the basic concepts of antibiotics and indications for antibiotic use, etc. The KAP questionnaire for the caregivers consisted of 30 structured or semi-structured questions, and is divided into four parts: (1) The demographics of the caregivers, including gender, age, educational level, family income, health insurance, and the relationship between the guardians and the children. (2) The knowledge part is to assess the guardian’s cognition of antibiotic-related knowledge, including the relationship between antibiotic use and drug resistance, and the indication of antibiotics. (3) The attitude part includes whether they agree that the use of antibiotics in advance can prevent diseases such as colds, whether children should get antibiotics immediately if they have respiratory tract infections, whether using antibiotics can speed up the recovery of colds and coughs, whether expensive antibiotics are better than cheap ones, and whether caregivers should gain more knowledge about antibiotics, etc. (4) The behavioral part evaluating the behavior of the guardians using antibiotics for their children includes whether they stored antibiotics that were needed by children at home, whether they used antibiotics that were stored at home or the remaining antibiotics, whether they changed the dosage of antibiotics according to the condition of children, and whether they took the initiative to ask doctors to prescribe antibiotics, etc. After the intervention, an evaluation component is added in the KAP questionnaire among the participants in the intervention group, which consists of 12 questions and is intended to evaluate whether the intervention population are satisfied with the intervention measures. Both of the data collection tools were developed after extensive literature searches of related studies and after consulting with experts on infectious diseases in children. All researchers will have a uniform training before the questionnaire is issued to be very familiar with each item in the questionnaire. Once the participants complete the questionnaire, they will be collected by the researchers and will be kept in good condition. We will screen and rule out invalid questionnaires in which the key information is incomplete and is filled out arbitrarily.

### 2.7. Data Analysis

After the questionnaires are collected and screened, two researchers will use the EpiData 3.1 database to double-enter the data and will then use SPSS19.0 for statistical analysis (SPSS Inc., Chicago, IL, USA). For the KAP questionnaire, we use frequency to analyze the demographic characteristics of the participants and the mean/median will be used to describe the overall score of each part. We set three options for the knowledge section: yes or no or do not know. If participants answer a question correctly, they get one point, however, if they answer incorrectly or do not know, they get 0 points. For the attitude and behavior section, we will utilize the Likert scale for the statistical analysis. Chi-square test will be applied to compare the knowledge, attitudes, and behavior differences of the guardians about antibiotics in different regions and to evaluate the knowledge, attitudes, and practice differences of the caregivers about antibiotics use for children among people with different demographic characteristics. For the knowledge retention questionnaire, in each section, we will calculate the percentage of each question that is answered correctly. Logistic regression and Pearson correlation coefficient will be used to determine the important factors affecting children’s knowledge of antibiotics, such as regions and school scale and so on.

### 2.8. Ethical Considerations

Before conducting this research, some ethical issues need to be considered. An informed consent form and other research-related documents were reviewed and approved by the Research Ethics Committee of Xi’an Jiaotong University (Ethical Code: 2018-451). We will obtain a written informed consent form from the participating children and guardians, in which the purpose of the study and their role in the study will be stated. The information will be stated verbally or written in a comprehensible way. There are two copies of the informed consent forms of which participants will keep one of them. They can also ask for details about the study and can make suggestions. It is voluntary to participate in the study and the participants are free to withdraw at any stage. Each participant will be allocated a unique identification number. We will inform participants that their information may be used for research purposes only and confidentiality will be guaranteed. All of the data will be stored in a safe place, accessible only to key researchers. Personally identifiable information such as the name or address of a participant will not published in any paper or documents. 

## 3. Discussion

As far as we know from our literature search, this school-level intervention is unique in China. It is the first educational intervention for both children and their caregivers to reduce the irrational use of antibiotics among children. Many studies in China have only concentrated on caregivers, and educational intervention studies targeting children are not well established. Furthermore, according to the results of the sixth census in China, there are a total of 145,790,011 children aged 5–14, which accounts for about 10.94% of China’s total population [47], hence our intervention targets both children and their caregivers. For caregivers, we expect to reduce the demand for prescribing antibiotics for children in upper respiratory tract infections and reduce self-medication for children. On the other hand, we hope that children will understand how to prevent infection and will establish an awareness of the rational use of antibiotics from childhood. If this research provides feasible positive results, we will extend the study to more schools so that more children and guardians can benefit from it. Furthermore, the implementation of such interventions can greatly enhance the rational use of antibiotics in children and prevent antibiotic resistance.

Children are the future users and prescribers of antibiotics, and so the rational use of antibiotics in children is not only significant to children, but also to their guardians. Both populations lack knowledge about antibiotics and, in addition, the easy accessibility of antibiotics without prescriptions is largely seen in China. Therefore, educating children and caregivers will be challenging but indispensable. During implementing the intervention study, we have difficulties to overcome. Firstly, because of the time-consuming nature of intervention studies, recruiting schools and participants is more difficult than for cross-sectional studies. Secondly, in theory, we hope that every participant in the intervention arm will complete all of the intervention measures, however, this is difficult to achieve. Besides, it is critical to control the contamination between the control group and the intervention group. Our study has some advantages. First, it is closely linked to the theme of antibiotic resistance, and we carry out an educational intervention about the rational use of antibiotics for children and caregivers from the school level to make up for the deficiencies in the domestic research in this area. Moreover, the content of our training sessions is not only limited to the rational use of antibiotics to treat infection, but it also focuses on how to prevent infection. Besides, we not only set a control group, we also compare the data before and after the intervention so that we can evaluate the net effect of the intervention. Last but not least, WeChat has become a way of life for the Chinese. People started to use it for text messaging, life moment sharing, and watching videos and so on. Data in 2016 showed that it attracted some 889 million monthly active users [48]. In our study, we will combine paper educational materials with network technology and we will implement interventions through the WeChat platform, therefore, the intervention measure in our research is novel. 

This study is with some deficiencies. The sample size is not large enough due to the heavy learning pressure, fewer primary schools in only one province are included, and rural primary schools are not included in our study, therefore generalization cannot be made. However, after this research, we hope to extend the intervention to more students and caregivers through the Ministry of Education. In addition, we cannot make sure that all of the participants will read the educational materials and will take part in the training sessions, which may have an effect on the final results. However, we will attempt to minimize this effect. Before the training session, each participant will receive a message reminding them to attend it on time and will be required to sign in. What is more, the content of the training session and the educational materials will be repeated. Lastly, the intervention period will only last for 3 months because of limited funding and time, and so further study will need to be conducted to observe the impact of long-term interventions.

## 4. Conclusions

This study aims to promote rational antibiotic use among caregivers and children in four primary schools in Baoji and Weinan. If the intervention measures show effectiveness, we will implement this intervention among large populations to benefit more children and caregivers. Besides, the findings of our study will be disseminated through different channels, for example, workshops, policy briefs, peer-reviewed academic papers in national and international journals, and presenting the findings in domestic and international conferences. At the same time, they will be used to inform the educational sector and policy-makers, hence regularly stimulating campaigns on the prudent use of antibiotics in primary schools. By doing so, all school-aged children can have a chance to attain knowledge about antibiotics from childhood and their awareness of the appropriate use of antibiotics can be established at a young age.
